# Device-Based Physical Activity Surveillance in Asian American Children and Adolescents: Participation and Activity Patterns in NHANES 2011–2014

**DOI:** 10.3390/children13091123

**Published:** 2026-08-22

**Authors:** Soyang Kwon, Pooja S. Tandon, Nilay S. Shah, Namratha R. Kandula

**Affiliations:** 1 Buehler Center for Health Policy and Economics, Feinberg School of Medicine, Northwestern University, Chicago, IL 60611, USA; 2 Seattle Children’s Research Institute, Seattle, WA 98121, USA; pooja.tandon@seattlechildrens.org; 3 Division of General Pediatrics, University of Washington Pediatrics, Box 356320, Seattle, WA 98115, USA; 4 Department of Medicine (Cardiology) and Preventive Medicine, Feinberg School of Medicine, Northwestern University, Chicago, IL 60611, USA; nilay.shah@northwestern.edu; 5 Department of Medicine (General Internal Medicine) and Preventive Medicine, Feinberg School of Medicine, Northwestern University, Chicago, IL 60611, USA; n-kandula@northwestern.edu

**Keywords:** a nationally representative sample, ActiGraph accelerometers, Asian race, youth, birthplace, acculturation

## Abstract

**Highlights:**

**What is the main finding?**

- Asian American children and adolescents had lower accelerometer-measured physical activity volume relative to age- and sex-specific national reference values.

**What is the implications of the main finding?**

- The consistently lower relative physical activity levels support the need for physical activity promotion efforts targeting Asian American children and adolescents.

**Abstract:**

Background: Asian American children and adolescents are underrepresented in physical activity research, and available evidence relies largely on self- or caregiver-reported measures. This study evaluated accelerometer-measured physical activity levels among Asian American children and adolescents, benchmarked against a national reference and explored differences by nativity and sex. Methods: We analyzed National Health and Nutrition Examination Survey (NHANES) 2011–2014 data from non-Hispanic Asian children and adolescents aged 6–17 years who provided valid wrist-worn ActiGraph accelerometer data. Nativity was classified as U.S.-born or non-U.S.-born. Physical activity was summarized using the Monitor-Independent Movement Summary (MIMS) units and sex- and age-specific MIMS percentiles using U.S. reference values. Survey-weighted linear regression models examined associations between nativity and MIMS percentile, adjusting for age group, sex, and family income. A sex-by-nativity interaction was tested. Results: Among 410 participants, the mean MIMS percentile was 36.7 (95% CI: 33.8–39.6), significantly below the national median. Overall MIMS percentiles did not differ by nativity. A sex-by-nativity interaction was observed but did not reach a statistical significance (*p* = 0.06). Descriptively, non-U.S.-born males tended to have higher MIMS percentiles than U.S.-born males (difference = 8.2; *p* = 0.16), whereas non-U.S.-born females tended to have lower MIMS percentiles than U.S.-born females (difference = −4.6; *p* = 0.19). Conclusions: Accelerometer-measured PA in Asian American children and adolescents was below the national reference. Nativity- and sex-related patterns were exploratory and warrant confirmation in larger samples.

## 1. Introduction

Regular physical activity (PA) is a key health behavior that supports physical and mental health across the life course [1,2,3]. Asian Americans are the fastest-growing major racial/ethnic group in the U.S. [4]. Available evidence indicates that Asian American adults engage in lower levels of PA compared with other major racial/ethnic groups [5,6] and that nativity and length of U.S. residence play important roles in shaping PA behavior and cardiometabolic risk among Asian Americans [7,8]. Yet, Asian Americans remain underrepresented in PA research [9,10].

Among Asian American children and adolescents, PA data are even more limited [11,12]. National survey data [13,14] indicated that Asian children and adolescents, females in particular, had the lowest prevalence of meeting aerobic PA recommendations (≥60 min/day) among major racial and ethnic groups. Studies examining the relationships between nativity, home language, acculturation, and PA in this population have produced inconsistent findings [13,15,16]. For example, Allen and colleagues [15] reported higher engagement in PA among U.S.-born Asian adolescents than their non-U.S.-born peers, whereas Unger and colleagues [16] found that greater acculturation to the U.S. was associated with less frequent PA participation. Our prior analysis using National Health and Nutrition Examination Survey (NHANES) 2011–2018 data found no clear association between language spoken at home and meeting aerobic PA recommendations among Asian American children and adolescents [13].

In evaluating PA among Asian American children and adolescents, most prior studies [13,14,15,16] have relied on self- or caregiver-reported PA, which is subject to recall and social desirability bias and may differentially misclassify PA across cultural contexts [17,18]. Wearable devices, such as research-grade ActiGraph accelerometers and consumer-grade Fitbit devices, capture body movement continuously and independently of a respondent’s PA perception or recall [19,20]. As national surveillance systems shift toward device-based measurement, the representativeness of the resulting estimates depends on who agrees to wear a device and who complies with the wear protocol [21,22]. Studies have reported that Asian Americans have historically been less willing to participate in health research [23,24], and that racial and ethnic minority groups are less likely to enroll in device-based assessment protocols and accumulate less wear time [22,25,26]. Whether these patterns extend to Asian American children and adolescents in device-based surveillance has not been examined. Systematic documentation of participation and compliance patterns is therefore essential to inform wearable deployment strategies in future research involving this population.

To address these gaps in understanding PA behaviors among Asian American children and adolescents, this study was guided by the social–ecological and immigrant health framework that recognizes intersecting influences of individual, family, sociocultural, and environmental contexts [27]. Nativity is conceptualized as a marker of migration-related context, reflecting potential differences in cultural norms, family expectations, language environment, and exposure to U.S. social and built environments [7,8]. The household language environment is one dimension of this context. These influences may operate differently by sex, particularly because gender norms in some Asian immigrant families may shape opportunities, encouragement, and perceived acceptability of sports and PA [28,29,30]. U.S. residence may also alter PA through changes in transportation patterns, school and neighborhood environments, and access to organized recreation [31,32,33].

The NHANES 2011–2014 cycles provide a unique opportunity to address these gaps through wrist-worn accelerometer data and the newly introduced Asian race category [34]. These data were used to establish national reference values for the device-independent acceleration metric, the Monitor-Independent Movement Summary (MIMS) [35], allowing PA levels to be interpreted against a population-referenced standard rather than in absolute units alone. Leveraging these data, the first aim of the present study was to evaluate accelerometer-measured PA among Asian American children and adolescents aged 6–17 years, expressed relative to national MIMS reference values. We hypothesized that mean MIMS percentile among Asian American children and adolescents would fall below the national MIMS reference median. The second aim was to examine whether their PA differed by nativity status (i.e., U.S.-born vs. non-U.S.-born). Given inconsistent findings in prior literature [13,15,16], no direction was hypothesized. Exploratory aims were to (1) examine whether the association between nativity status and PA varied by sex and to (2) characterize their participation in device-based PA surveillance across two sequential stages: (a) the probability of contributing an accelerometer data file among eligible participants, and (b) the probability of meeting valid wear-time criteria among those who contributed a file.

## 2. Methods

### 2.1. Participants

We analyzed publicly available NHANES 2011–2012 and 2013–2014 data. NHANES is a continuous, cross-sectional survey of the noninstitutionalized U.S. population using a complex, multistage probability sampling design. Beginning in 2011, NHANES introduced a standalone Asian race category and oversampled Asian participants [34]. The analytic sample of this study included non-Hispanic Asian participants (“Asian” hereafter) aged 6–17 years. For exploratory aim 2, non-Hispanic White participants (“White” hereafter) aged 6–17 years served as a comparison group. The study was determined to be exempt from review by the Institutional Review Board of Northwestern University (STU00222504).

### 2.2. Data Collection

NHANES 2011–2014 administered a demographic interview and device-based PA measurement (PAM) using the ActiGraph GT3X+ (Ametris, Pensacola, FL, USA) at 80 Hz. Prior pediatric studies have supported its validity for wrist-based PA assessment [36,37,38]. During the Mobile Examination Center visit, the device was placed on the participant’s non-dominant wrist using a mesh band. Participants were instructed to wear the device 24 h a day for seven full consecutive days and return it via mail.

### 2.3. Measures

Nativity status was determined by self- or caregiver-reported country of birth and categorized as U.S.-born or non-U.S.-born. Language spoken at home was categorized as mostly English, English and non-English equally, or mostly non-English.

Daily MIMS was used to evaluate PA levels. We downloaded publicly available NHANES 2011–2014 PAM datasets containing acceleration data in MIMS units from the NHANES website. MIMS is a non-proprietary, open-source, device-independent metric derived from raw triaxial acceleration data and designed to ensure data comparability across different accelerometer models [39]. Valid wear minutes, including both wake and sleep wear minutes, were extracted based on the wear status variable provided in the datasets [40]. A day with at least 600 wear minutes was considered as a valid wear day, consistent with prior analyses of NHANES 2011–2014 wrist accelerometer data [41,42,43] and with another study of children using a 24-hour wrist-wear protocol [44]. Participants were included in the final analysis if they provided at least three valid wear days.

To contextualize PA levels among Asian American children and adolescents relative to their peers, we used sex- and age-specific MIMS percentiles based on the national MIMS reference values [35]. These reference percentiles were previously developed by combining wrist-worn accelerometer data from NHANES 2011–2014 and the 2012 National Youth Fitness Survey, calculating each participant’s mean daily MIMS during wake and sleep periods across valid wear days, and applying Generalized Additive Models for Location, Scale, and Shape (GAMLSS) to generate smoothed age- and sex-specific percentile curves and tables across the lifespan [35]. The reference values included MIMS accumulated during both waking and sleeping wear, consistent with our definition of valid wear, which counted wear during both periods. These MIMS reference values are not directly comparable with previously published references based on activity counts [45] or Euclidean Norm Minus One (ENMO) [46]. For the present study, each participant’s mean daily MIMS across valid wear days was converted to a percentile using the corresponding sex- and age-specific GAMLSS distribution parameters. No interpolation between ages was required because separate model parameters were available for each single year of age from 3 to 19 years.

Covariates included age group (6–11 vs. 12–17 years), sex, and poverty index ratio categorized as low (<1.0; below poverty), middle (1.0 to <3.0), or high (≥3.0).

### 2.4. Analysis

All statistical analyses were performed using SAS (version 9.4; SAS Institute Inc, Cary, NC, USA), accounting for NHANES weights, strata, and primary sampling units. The 2011–2012 and 2013–2014 cycles were combined in accordance with NHANES analytic guidelines for multi-cycle estimation, with four-year sample weights constructed by halving the corresponding two-year MEC examination weights (WTMEC2YR/2), and masked variance units (SDMVSTRA, SDMVPSU) specified. Estimates for the analytic subgroup were obtained using domain analysis (SAS PROC SURVEYREG with a DOMAIN statement) rather than by subsetting the data file, so that variance estimation retained the full sample design.

Descriptive statistics were calculated for all study variables. To assess potential selection bias, we compared demographic characteristics between participants included in the analytic sample and those excluded using Rao-Scott chi-square tests.

For the first aim, we calculated the mean and standard error of MIMS percentile. For the second aim, multivariable linear regression models were used to compare MIMS percentiles between U.S.-born (reference group) and non-U.S.-born participants, adjusting for age group (12–17 years vs. 6–11 years [reference group]), sex (males vs. female [reference]), and family income (low, middle vs. high [reference group]). Although MIMS percentiles are derived with continuous age adjustment, we additionally included age group as a covariate to allow for the possibility that the association between nativity and relative PA differs by developmental stage within this population. Family income was modeled categorically rather than continuously to avoid imposing a linear form on the income–PA association and to preserve interpretability (e.g., below poverty). Language spoken at home was not included in the models because it was considered an alternative indicator of immigration-related context that conceptually overlapped with nativity-related measures. Instead, we explored differences in MIMS percentiles by language spoken at home as a sensitivity analysis. Because language spoken at home was missing for 109 participants, those participants were excluded from this analysis without imputation (complete-case analysis).

For exploratory aim 1, an interaction term between sex and nativity was added to assess whether the nativity-PA association differed by sex. Sex-stratified analyses were also conducted to assess the direction and the magnitude of the association within each sex. Additionally, we tested an interaction between age group and nativity and conducted age group–stratified analyses.

For exploratory aim 2, we compared Asian and White participants on two sequential outcomes: the proportion contributing an accelerometer data file among all eligible participants, and, among those who contributed a file, the proportion meeting valid wear-time criteria. Design-adjusted Rao–Scott chi-square tests were used for categorical variables and design-adjusted t-tests were used for continuous variables. Model diagnostics were assessed using residual versus fitted and normal Q-Q plots. Multicollinearity and formal global model-fit statistics were not separately evaluated.

## 3. Results

The NHANES 2011–2014 examinations included 494 Asian participants aged 6–17 years and 1109 White participants. Of these, 434 Asian participants (87.8%) and 1013 White participants (91.1%) received an accelerometer and provided an accelerometer data file (*p* = 0.07; Table 1). Among those with available files, 410 Asian American participants (93.9%) and 949 White participants (93.8%) provided at least three valid days of accelerometer data (*p* = 0.94). Among Asian participants, demographic characteristics (e.g., sex, age, family income, and nativity) did not significantly differ between 410 with sufficient accelerometer data and 84 without (*p* > 0.10; Supplementary Table S1).

As shown in Table 2, of the 410 participants who provided valid accelerometer data, 292 (70.3%) were U.S.-born and 118 (29.7%) were non-U.S.-born. Participants with and without missing data on language spoken at home had similar distributions of sex, age group, and family income, but missingness was more common among U.S.-born than non-U.S.-born participants (26.4% [88/292] in U.S.-born group vs. 14.6% [21/118] in the non-U.S.-born group).

The overall mean MIMS percentile was 36.7 (95% CI = 33.8, 39.6; Table 3), significantly lower than the national median (50th percentile; *p* < 0.01). In sex-combined analysis, MIMS percentiles did not differ by nativity (36.2 [95% CI = 33.3, 39.1] for the U.S.-born vs. 37.8 [95% CI = 31.6, 44.0] for the non-U.S.-born). Within the non-U.S.-born group, males had significantly higher mean MIMS percentiles than females (*p* = 0.01), whereas this sex difference was not observed within the U.S.-born group (*p* = 0.73).

A multivariable linear regression model indicated a marginal nativity-by-sex interaction (*p* = 0.06; Table 4). Model diagnostics showed no substantial evidence of nonlinearity or heteroscedasticity. Sex-stratified estimates suggested differing patterns by sex: non-U.S.-born males tended to have higher MIMS percentiles than U.S.-born males (difference = 8.2; *p* = 0.16), whereas non-U.S.-born females tended to have lower MIMS percentiles than U.S.-born females (difference = −4.6; *p* = 0.19).

When associations between language spoken at home and MIMS percentile were examined by sex, similar patterns were observed, although the differences were not statistically significant (Supplementary Table S2). Among males, those who reported speaking only non-English or more non-English at home tended to have higher MIMS percentiles than those who reported speaking English only or more English (difference = 8.0; *p* = 0.28), as did those who reported speaking English and non-English equally (difference = 3.9; *p* = 0.22). In contrast, among females, those who reported speaking only non-English or more non-English (difference = −5.1; *p* = 0.14) or speaking English and non-English equally (difference = −3.8; *p* = 0.07) tended to have lower MIMS percentiles than those who reported speaking English only or more English. No significant interaction between age group and nativity was observed (Supplementary Table S3).

## 4. Discussion

Using NHANES 2011–2014 accelerometer data, we found that mean PA levels among Asian American children and adolescents aged 6–17 years ranked below the national median. Overall PA levels did not differ by nativity status, though a borderline interaction between nativity and sex was observed (*p* = 0.06). Specifically, non-U.S.-born males tended to have higher MIMS percentiles than U.S.-born males, whereas non-U.S.-born females tended to have lower MIMS percentiles than U.S.-born females, although these differences did not reach statistical significance in stratified models. In addition, Asian American children and adolescents showed a lower probability of contributing an accelerometer data file than White children and adolescents, although the difference did not reach conventional statistical significance (*p* = 0.07); however, wear-time compliance was comparable among those with an available file.

**Physical activity levels in Asian American children and adolescents.** Our findings confirm that using accelerometer-measured PA data, Asian American children and adolescents engaged in lower levels of PA relative to their U.S. peers, consistent with previous research utilizing self-reported measures [13,14,15,47,48] as well as the same NHANES accelerometer data [49,50]. The absence of sex difference in average MIMS percentiles suggests that male and female Asian American children and adolescents exhibit comparably low PA levels relative to their peers, underscoring the need for PA promotion efforts targeting Asian American children and adolescents of both sexes. Importantly, this study contributes to the literature by quantifying these disparities using accelerometer-derived MIMS percentiles, demonstrating that Asian American children and adolescents averaged around the 37th percentile relative to the national median (50th percentile).

**Nativity and physical activity levels.** We observed potential sex-specific patterns in the association between nativity and PA among Asian American children and adolescents. We also found similar sex-specific patterns in the association between language spoken at home and PA. However, the sex-by-nativity interaction was borderline, and stratified estimates were imprecise and non-significant due to the limited statistical power; these patterns should therefore be interpreted as hypothesis-generating and require confirmation in larger samples. Among non-U.S.-born participants, females were less active than males, whereas this difference was not evident among U.S.-born youth. This pattern is broadly consistent with reports that gendered expectations regarding sports participation persist in some immigrant families and may attenuate with greater exposure to the U.S. context [28,29,30]. Given the imprecision of the stratified estimates, however, we cannot distinguish this interpretation from chance variation. If replicated in larger samples, such patterns would support culturally adapted approaches to PA promotion among Asian American youth.

**Inclusion of Asian American children and adolescents in wearables research.** Growing evidence suggests that engagement in device-based PA research is not evenly distributed across sociodemographic groups. In a large population-based study, Kim et al. [22] reported that children from racial and ethnic minority groups were less likely to participate in a research study involving wearable devices, highlighting barriers to engagement in such research activities. Our pilot study [51] also showed that although Asian parents were willing to complete surveys, 16% of them declined their child’s participation in accelerometer wear. Consistent with these patterns, the present study found that Asian American children and adolescents were less likely to contribute accelerometer data files than their White American counterparts, although the difference did not reach conventional statistical significance (*p* = 0.07). Our findings also showed that among participants with an available accelerometer file, Asian American children and adolescents had wear-time compliance comparable to that of White children and adolescents. Together, these findings indicate that disparities may arise primarily at earlier stages of participation rather than during wear-time compliance, although the specific stage of participation (e.g., invitation, consent, device acceptance, or device return) cannot be determined from the available NHANES documentation. Taken alongside prior reports of lower enrollment among racial and ethnic minority groups in device-based protocols [22,25,26], our findings support intentional efforts to improve the inclusion of Asian American children and adolescents in device-based surveillance and intervention research.

**Public Health Implications.** The consistently lower relative PA levels observed among Asian American children and adolescents highlight the need for targeted PA promotion strategies within this population. The borderline nativity–sex interaction raises the possibility that such strategies may need to account for the intersecting influences of nativity and sex; however, these findings are hypothesis-generating rather than confirmatory. In addition, developing appropriately tailored PA intervention strategies will require surveillance data disaggregated by Asian origin, as aggregate estimates may obscure subgroup differences directly relevant to intervention design.

**Strengths and Limitations.** The primary strength of this study is the utilization of high-resolution free-living accelerometer data, which reduced recall and social desirability biases inherent in self-reported PA measures. This approach provided a more accurate quantification of PA levels in Asian American children and adolescents. In addition, this study is among the few to evaluate PA in a national sample of Asian American children and adolescents.

Several limitations warrant consideration. First, lower participation rates in Asian individuals could potentially have influenced estimates of PA if participation was associated with PA levels, although the direction of such bias is uncertain. Second, sample sizes for individual Asian origin groups (e.g., Chinese, Indian, Filipino) were too small to support origin-specific analyses. As a result, the pan-Asian category used in this study aggregated diverse populations, potentially masking heterogeneity in PA patterns across Asian American communities. Therefore, the inability to disaggregate is a major constraint on both internal validity and public-health translation. Third, as this study used data collected over a decade ago, population-level PA behaviors may have changed over time. However, these cycles represent the most recent NHANES data with device-based accelerometer measurements. Fourth, the translation of MIMS units into moderate- and vigorous-intensity PA thresholds remains unestablished, hindering our ability to compare these findings against current aerobic PA guidelines. Fifth, MIMS percentiles reflect total 24-hour movement, including both waking and sleeping periods; therefore, observed differences may reflect variation in waking activity, sleep duration, nocturnal movement, or a combination of these factors. Sixth, nativity as measured here does not capture age at migration, duration of U.S. residence, immigrant generation, or country of origin. Seventh, because several exploratory interaction and stratified analyses were conducted without adjustment for multiple comparisons, these findings may be vulnerable to type I error and should be considered hypothesis-generating. The sample size was limited to provide sufficient statistical power for interaction and stratified analyses. Finally, residual confounding by unmeasured factors (e.g., sleep duration) may have influenced the findings.

In conclusion, this study provides device-based evidence that Asian American children and adolescents engage in significantly lower PA levels than U.S. peers. Although overall PA levels did not differ by nativity, the borderline interaction between nativity and sex generated a hypothesis that migration-related factors influence PA differently for male and female Asian children and adolescents.

## Figures and Tables

**Table 1:** Accelerometer data availability of Asian American children and adolescents relative to White American children and adolescents in NHANES 2011–2014.

	Asian	White	*p*-Value
Eligible for NHANES examination, unweighted *n*	494	1109	NA
Accelerometer data files available, unweighted *n* (weighted %)	434 (87.8)	1013 (91.1)	0.07
The number of valid wear days, weighted mean (SE) ^a^	6.2 (0.1)	6.2 (0.1)	0.82
Participants with sufficient accelerometer wear, unweighted *n* (weighted %) ^a,b^	410 (93.9)	949 (93.8)	0.94
Wear hours per day, weighted mean (SE) ^c^	21.9 (0.2)	21.9 (0.1)	0.94

^a^ Analyses included participants with available accelerometer data files; ^b^ Having at least three valid wear days was defined as sufficient accelerometer wear; ^c^ Analyses included participants with sufficient accelerometer data; NA, not applicable; SE, standard error.

**Table 2:** Characteristics of Asian American children and adolescents in NHANES 2011–2014.

Variable	U.S.-Born (*n* = 292)	Non-U.S.-Born (*n* = 118)
	Unweighted *n* (weighted %)	Unweighted *n* (weighted %)
Sex		
Male	149 (52.6)	51 (44.7)
Female	143 (47.4)	67 (55.3)
Age group		
6–11 years	154 (51.6)	41 (34.3)
12–17 years	138 (48.4)	77 (65.7)
Family income		
Low (below poverty)	25 (7.2)	25 (22.2)
Middle	131 (45.7)	41 (33.4)
High	136 (47.1)	52 (44.4)
Language spoken at home		
Only English or more English than non-English	118 (59.6)	38 (41.2)
English and non-English equally	53 (25.0)	17 (17.2)
Only non-English or more non-English than English	33 (15.4)	42 (41.6)
Missing ^a^	88	21

^a^ A total of 109 participants (22.9%) who did not report language spoken at home were not included in weighted % calculation.

**Table 3:** Comparisons of MIMS and MIMS percentiles between U.S.-born and non-U.S.-born Asian American children and adolescents.

	All (*n* = 410)	U.S.-Born (*n* = 292)	Non-U.S.-Born (*n* = 118)
Daily MIMS	Weighted mean (95% CI)	Weighted mean (95% CI)	Weighted mean (95% CI)
All	15,083 (14,716, 15,451)	15,236 (14,789, 15,683)	14,723 (13,928, 15,517)
By sex			
Male	15,114 (14,572, 15,655)	14,976 (14,300, 15,652)	15,525 (14,922, 16,127)
Female	15,052 (14,537, 15,568)	15,525 (14,922, 16,127)	14,098 (13,367, 14,828)
By age group			
6–11 years	17,351 (16,716, 17,985)	17,217 (16,412, 18,022)	17,827 (16,924, 18,731)
12–17 years	13,116 (12,544, 13,687)	13,122 (12,526, 13,717)	13,105 (11,972, 14,237)
MIMS percentile			
All	36.7 (33.8, 39.6)	36.2 (33.3, 39.1)	37.8 (31.6, 44.0)
By sex			
Male	38.8 (35.0, 42.5)	36.6 (32.8, 40.5)	44.7 (34.8, 54.5)
Female	34.6 (31.2, 38.0)	35.7 (31.8, 39.6)	32.3 (27.2, 37.4)
By age group			
6–11 years	34.1 (30.1, 38.2)	32.9 (27.7, 38.0)	38.7 (31.6, 45.9)
12–17 years	38.8 (34.6, 43.1)	39.7 (35.7, 43.8)	37.3 (29.3, 45.4)

CI, confidence interval; MIMS, Monitor-Independent Movement Summary.

**Table 4:** Multivariable linear regression models to compare MIMS percentiles between U.S.-born and non-U.S.-born Asian American children and adolescents.

Predictor	Combined (*n* = 410)			Males (*n* = 200)			Females (*n* = 210)		
	β ± SE	*p*-Value	95% CI	β ± SE	*p*-Value	95% CI	β ± SE	*p*-Value	95% CI
Intercept	34.4 ± 3.1	<0.01	28.2, 40.7	36.1 ± 3.3	<0.01	29.2, 42.9	32.9 ± 3.8	<0.01	25.2, 40.6
Sex: male vs. female	0.5 ± 2.7	0.84	−4.9, 6.0	NA	NA	NA	NA	NA	NA
Age group: 12–17 vs. 6–11 years	5.0 ± 3.0	0.10	−1.0, 11.1	6.0 ± 4.3	0.17	−2.7, 14.7	4.5 ± 4.1	0.28	−3.9, 12.9
Family income: low vs. high	−2.8 ± 4.2	0.51	−11.3, 5.8	−9.1 ± 7.0	0.20	−23.3, 5.1	2.4 ± 4.9	0.63	−7.7, 12.5
Family income: middle vs. high	−1.5 ± 2.8	0.60	−7.3, 4.3	−4.3 ± 3.9	0.28	−12.3, 3.7	1.3 ± 3.9	0.73	−6.6, 9.3
**Nativity:** non-U.S.-born vs. U.S.-born	−4.4 ± 3.2	0.18	−11.0, 2.1	**8.2 ± 5.7**	**0.16**	**−3.4, 19.8**	**−4.6 ± 3.4**	**0.19**	**−11.6, 2.4**
**Sex × Nativity**	**11.9 ± 6.1**	**0.06**	−0.6, 24.4	NA	NA	NA	NA	NA	NA

The exposures of interest are shown in bold; β ± SE, weighted coefficient ± standard error; CI, confidence interval; MIMS, Monitor-Independent Movement Summary; NA, not applicable.

## Data Availability

Data are available on the NHANES website. https://www.cdc.gov/nchs/nhanes/index.html (accessed on 20 August 2026).

## Supplementary Materials

The following supporting information can be downloaded at https://www.mdpi.com/article/10.3390/children13091123/s1, Table S1: Comparison of demographic characteristics between included and excluded participants in the NHANES 2011–2014 MEC sample of Asian children and adolescents aged 6–17 years; Table S2: Multivariable linear regression models to compare MIMS percentiles between U.S.-born and non-U.S.-born Asian American children and adolescents by sex; Table S3: Multivariable linear regression models to compare MIMS percentiles between U.S.-born and non-U.S.-born Asian American children and adolescents by age group.

## Abbreviations

MIMS	Monitor-Independent Movement Summary
NHANES	National Health and Nutrition Examination Survey
PA	Physical activity
PAM	Physical Activity Monitor
