# Supplementary material for: Device-Based Physical Activity Surveillance in Asian American Children and Adolescents: Participation and Activity Patterns in NHANES 2011–2014
Source: Children (Basel). 2026 Aug 22;13(9):1123. doi: 10.3390/children13091123 (PMC13605227; doi:10.3390/children13091123)
Supplement: Supplementary file 1 [file children-13-01123-s001.zip › children-4461447-supplementary.pdf]

Supplementary Data

Supplementary Table S1. Comparison of demographic characteristics between included and excluded participants in the NHANES 2011–2014 MEC sample of Asian children and adolescents aged 6–17 years

Variable	Included (n=410)	Excluded (n=84)	p-value[a]
	Unweighted n (weighted %)	Unweighted n (weighted %)	
Sex			0.22
Male	200 (50.3)	47 (56.5)	
Female	210 (49.7)	37 (43.5)	
Age group			0.49
6-11 years	195 (46.5)	43 (49.9)	
12-17 years	215 (53.5)	41 (50.1)	
Family income			0.14
Low (below poverty)	50 (11.7)	7 (7.7)	
Middle	172 (42.0)	45 (52.7)	
High	188 (46.3)	32 (39.6)	
Nativity			0.76
U.S.-born	292 (70.3)	60 (68.5)	
Non-U.S.-born	118 (29.7)	24 (31.5)	

NHANES, National Health and Nutrition Examination Survey; MEC, Mobile Examination Center.

[a]P-values were derived from Rao-Scott chi-square tests.

Supplementary Table S2. Multivariable linear regression models to compare MIMS percentiles between U.S.-born and non-U.S.-born Asian American children and adolescents by sex

	Males (n=145)		Females (n=156)	
Predictor	β±SE	p-value	β±SE	p-value
Intercept	34.4±1.4	<0.01	32.9±1.6	<0.01
Age group: 12-17 vs. 6-11 years	9.0±2.5	<0.01	5.7±0.5	<0.01
Family income: low vs high	-11.7±3.6	<0.01	3.1±0.8	<0.01
Family income: middle vs high	-6.0±3.4	0.09	-0.3±2.2	0.91
**Language spoken at home:** Only non-English or more non-English than English vs. Only English or more English than non-English	**8.0±7.2**	**0.28**	**-5.1±3.4**	**0.14**
**Language spoken at home:** English and non-English equally vs. Only English or more English than non-English	**3.9±3.1**	**0.22**	**-3.8±2.0**	**0.07**

One hundred and nine participants were excluded from these analyses due to missing data on language spoken at home; The exposures of interest are shown in bold.
β±SE, weighted coefficient±standard error; MIMS, Monitor-Independent Movement Summary; NA, not applicable.

Supplementary Table S3. Multivariable linear regression models to compare MIMS percentiles between U.S.-born and non-U.S.-born Asian American children and adolescents by age group

Predictor	Combined (n=410)		Age 6-11 years (n=195)		Age 12-17 years (n=215)	
	β±SE	p-value	β±SE	p-value	β±SE	p-value
Intercept	31.8±3.3	<0.01	32.3±4.0	<0.01	37.7±3.7	<0.01
Sex: male vs. female	3.8±2.1	0.08				
Age group: 12-17 vs. 6-11 years	4.8±3.6	0.07	NA	NA	NA	NA
Family income: low vs high	-1.9±4.4	0.66	1.2±6.8	0.86	-4.7±4.2	0.27
Family income: middle vs high	-1.4±3.0	0.64	-2.5±3.4	0.46	-0.8±4.2	0.86
**Nativity:** non-U.S.-born vs. U.S.-born	5.8±4.8	0.24	**5.5±4.8**	**0.26**	**-1.0±4.3**	**0.82**
**Age group*Nativity**	**-7.6±6.1**	**0.22**	NA	NA	NA	NA

The exposure of interest is shown in bold.
β±SE, weighted coefficient±standard error; MIMS, Monitor-Independent Movement Summary; NA, not applicable.
